# Antiglycation Activities and Common Mechanisms Mediating Vasculoprotective Effect of Quercetin and Chrysin in Metabolic Syndrome

**DOI:** 10.1155/2020/3439624

**Published:** 2020-07-27

**Authors:** Osama A. A. Ahmed, Ahmad S. Azhar, Mayada M. Tarkhan, Khadijah S. Balamash, Hany M. El-Bassossy

**Affiliations:** ^1^Department of Pharmaceutics, Faculty of Pharmacy, King Abdulaziz University, Jeddah, Saudi Arabia; ^2^Department of Pharmaceutics and Industrial Pharmacy, Faculty of Pharmacy, Minia University, Minia, Egypt; ^3^Pediatric Cardiac Center of Excellence, Faculty of Medicine, King Abdulaziz University, Jeddah, Saudi Arabia; ^4^Department of Biochemistry, Faculty of Science, King Abdulaziz University, Jeddah, Saudi Arabia; ^5^Department of Pharmacology and Toxicology, Faculty of Pharmacy, Zagazig University, Zagazig, Egypt

## Abstract

Multiple risk factors combine to increase the risk of vascular dysfunction in patients suffering from metabolic syndrome (MetS). The current study investigates the extent to which quercetin (Q) and chrysin (CH) protect against vascular dysfunction in MetS rats. MetS was induced by feeding rats a high-salt diet (3%) and fructose-enriched water (10%) for 12 weeks. Thoracic aorta was isolated from MetS rats and from control rats, with the latter being injured by methylglyoxal (MG). Aortae were incubated with CH and Q, and vascular reactivity was evaluated through the analysis of aortic contraction and relaxation in response to PE and ACh, respectively. The formation of advanced glycation end products (AGEs) and the free radical scavenging activity of 1,1-diphenyl-2-picrylhydrazyl (DPPH) were also evaluated following the introduction of CH and Q. The increased vasoconstriction and impaired vasodilation in MetS aortae were significantly ameliorated by Q and CH. Similarly, they ameliorated glycation-associated exaggerated vasoconstriction and impaired vasodilation produced by MG in control aortae. In addition, both Q and CH were effective in reducing the formation of AGEs and inhibition of glycosylation in response to MG or fructose treatment. Finally, Q successfully scavenged DPPH free radicals while CH showed significant vasodilation of precontracted aorta that was inhibited by L-NAME. In conclusion, Q and CH provide protection against vascular dysfunction in MetS by interfering with AGEs formations and AGEs-associated vascular deterioration, with CH being largely dependent on NO-mediated mechanisms of vasodilation.

## 1. Introduction

The condition “metabolic syndrome (MetS),” describes a group of conditions including central obesity, dyslipidaemia, hyperglycaemia, and hypertension. The presence of all of the mentioned conditions, rather than only a few, is required in order to draw a conclusive diagnosis of MetS [1]. CVD and type 2 diabetes mellitus (T2DM) develop more commonly in individuals who have any of these interrelated metabolic risk factors [2]. The effect of the syndrome upon the population is significant, and as the incidence of MetS is rising, it is important to devise well-defined diagnostic criteria and treatment guidelines.

It is estimated that 37% of adults in the Kingdom of Saudi Arabia have MetS. The prevalence of the syndrome is lower in rural areas than urban populations, reflecting lifestyle differences in the levels of activity between the two populations [3]. In particular, the rising obesity trend, especially central obesity, is considered a key factor in the development of MetS [4]. Central obesity is associated with glucose intolerance, in which the body is less able to use glucose [5]. In MetS, an insulin-resistant state develops, ultimately leading to hyperglycaemia [6].

The adverse side effect of hyperglycaemia upon the cardiovascular system comes from the frequent accompaniment of impaired fibrinolytic pathways and hypercoagulation [7]. Moreover, in MetS, persistent hyperglycaemia promotes glycation, the nonenzymatic reaction between monosaccharide sugars and protein. The glycation of proteins is further heightened by increased quantities of the reactive sugar derivative, methylglyoxal (MG), which is also elevated secondary to hyperglycaemia. This reaction is considered irreversible, producing compounds known as AGEs [8]. AGEs cause blood vessels to become rigid, which, when combined with other pathological manifestations of diabetes, leads to persistent microvascular complications [9, 10]. Furthermore, as an adjunct to the production of AGEs, the glycoxidation products, dityrosine and N′-formyl kynurenine, are formed. These are useful markers that can be quantified to determine the degree of oxidative protein damage [11, 12].

Hyperglycaemia is not the only MetS condition that is damaging to the vasculature. Persistent hypertension and hyperlipidaemia also contribute to increased inflammation and oxidative stress and reduced production of the vasodilator, nitric oxide (NO) [13, 14]. The adverse side effects to vessel function manifest themselves as an attenuation of vasodilation and an increase in vasoconstriction [15]. A number of synthetic pharmaceuticals are used to attenuate vascular dysfunction, but their side effects are undesirable; this highlights the need to identify other effective compounds that are therapeutically safe and do not carry the same adverse side effects. To this end, researchers have investigated a number of naturally occurring compounds, many of which are flavonoids [16].

Two such examples are the flavonol quercetin (Q) and the flavone chrysin (CH). Consistent with other flavonoids, both compounds bear the distinctive tricyclic polyphenolic structure [17, 18]. Q is a ubiquitous compound, found in many fruits and vegetables including apples, peppers, and onions. On the other hand, sources of CH are less common but include chamomile, honey, and passionflower (*Passiflora caerulea*) [18, 19]. Q is reported to offer diverse health benefits, such as anti-inflammation, antioxidation, and the ability to stimulate endothelial production of NO [20–22]. Such reactions are extremely valuable in the prevention and even treatment of grave conditions such as cancer and the wide array of disorders that make up cardiovascular diseases. The established beneficial effects of Q offer a strong basis on which the current research can build. Based on such findings, Q is a potential compound that could be used to reduce MetS-initiated vascular damage. Similar to Q, CH has also been described as offering antioxidant and anti-inflammatory benefits which, as mentioned before, are key reactions involved in the prevention of many diseases. Such benefits of both Q and CH have been strongly established, and the results obtained conclude a significantly desirable effect, rather than weak conclusions based on speculation. It must be said however that research indicates that CH has less pronounce effects when compared to Q [23, 24]. Therefore, this study aims to explore the extent to which CH and Q offer vascular protection to MetS aorta and the potential mechanisms behind any effects observed.

## 2. Materials and Methods

### 2.1. Drugs and Chemicals

The following compounds were purchased from Sigma-Aldrich, Dorset, UK: acetylcholine (ACh), aminoguanidine (AG), bovine serum albumin (BSA), chrysin (CH), cinnamaldehyde (CA), diphenyl-2-picrylhydrazyl (DPPH), ferulic acid (Fer), fructose (F), methylglyoxal (MG), N_*ω*_-Nitro-L-arginine methyl ester hydrochloride (L-NAME), phenylephrine (PE), and quercetin (Q). CH, Q, and DPPH were dissolved in dimethyl sulfoxide (DMSO) at a concentration of 0.1% of the reaction media; all other compounds, which were of analytical grade, were dissolved in ultrapure deionized water.

### 2.2. Experimental Animals

Male Wistar rats aged 7 weeks and weighing between 180 and 200 g were obtained from King Fahd Medical Research Center (King Abdulaziz University, Jeddah, Saudi Arabia). Groups of four rats were kept in polypropylene animal housing; adequate ventilation, purified water, and standard rodent diet pellets were constantly available. Approval for the study's experimental protocol was granted by the Research Ethical Committee, Faculty of Pharmacy, King Abdulaziz University, Jeddah, Saudi Arabia (approval number 1071439). The Saudi Arabia Research Bioethics and Regulations were observed throughout the study.

To induce MetS, for 12 weeks, the 30 rats were given fructose-enriched water (10%) and high-salt diet (3%) [25] while unadulterated food and water were given to control rats. Weight of MetS rats was 250 g +.

### 2.3. Study Protocol

The rats were randomly allocated to either the control (C) or MetS group. After 12 weeks, the rats were decapitated by rodent guillotine and their descending thoracic aortae harvested. Termination of the rats by guillotine was necessary in order to avoid blood clotting inside the aorta that affects the current experiments.

### 2.4. Measurements of MetS Indices

Systolic blood pressure (SBP) was measured in control and in rats fed high-fructose and high-salt diet at the end of study by tail cuff method as described in detail in previous work of our laboratories [26]. Briefly, the measurement is preceded by an equilibrating 5–10 min period for the rats in the warming chamber, followed by 10 repetitions of the automated inflation-deflation cycles. Serum insulin level was determined using enzyme-linked immunosorbent assay kit using antirat insulin antibodies (Millipore, Billerica, Massachusetts).

### 2.5. Effect of Q and CH on MetS Vascular Dysfunction

To determine vascular reactivity, previously established techniques were used to isolate the aorta [26, 27]. Excised aortae from MetS animals were defatted, sectioned into 3 mm rings, and then suspended in an automated organ bath (Panlab, Barcelona, Spain). In addition to containing a single section of aorta, each channel contained 25 ml of Krebs Henseleit buffer (118 mM NaCl, 4.8 mM KCl, 2.5 mM CaCl_2_, 1.2 mM MgSO_4_, 1.2 mM KH_2_PO_4_, 25 mM NaHCO_3_, and 11.1 mM glucose). The bath was incubated at 37°C and continuously aerated with a 95% O_2_ and 5% CO_2_ gas mixture. Every 30 min, the buffer solution was replaced. To measure aortic tension, an isometric force transducer (ADInstruments, Bella Vista, Australia) was used. Data were collected and recorded using the PowerLab Data Interface Module; this was connected to a PC operating LabChart software v8 (ADInstruments).

To achieve the equilibrium state, aortic tension was adjusted to 1500 mg ± 50 for 20 min. The primary initiator of aortic contraction was PE (10^−5^ M), and relaxation was stimulated by ACh (10^−5^ M). Once resting-state tension was achieved (1500 mg ± 50), Q and CH at concentrations 10 and 30 *μ*M were added to the channels and then incubated at 37°C for 60 min. The vehicle (0.1% DMSO) was added to the control channels. To study the contractile response of the aorta following incubation, PE (10^−8^ to 10^−5^ M) was cumulatively added. The similar cumulative addition process was applied using ACh (10^−8^ to 10^−5^ M) to evaluate the relaxation response but in submaximal PE-precontracted vessels. To establish contraction, tension increase was measured in mg, and, conversely, the percentage of the PE-induced contraction was used to establish relaxation.

### 2.6. Effect of Q and CH on MG-Induced Vascular Dysfunction

For the MG experiment, the same procedure was followed, except that the aorta rings were isolated from control animals and suspended. Then aortic contraction and relaxation were initially stimulated by PE and ACh, respectively, before being incubated with MG (100 *μ*M) in absence or presence of Q or CH (10 and 30 *μ*M) at resting tension (1500 mg ± 50) for 60 min. Then, as previously described, cumulative concentrations of PE and ACh (10^−8^ to 10^−5^ M) were added.

### 2.7. Effect of Q and CH on AGE Production

96-well black plate was used to evaluate the effect of Q and CH on the formation of AGEs [28]. Bovine serum albumin (BSA), 10 mg/ml in phosphate buffer saline (PBS), was added first to the wells, followed by the addition of Q and CH (10–100 *μ*M). AG (1 mM) was used as a glycation inhibitor and was added to the wells containing the BSA and compounds. To this mixture, MG (50 mM) was added directly after preparation and left for incubation in the dark at 37°C for 1 h. The same was done with fructose (50 mM) except that the incubation time was 2 weeks and sodium azide 0.02% was added to all of the wells. A row of wells was kept as control where PBS was added instead of MG or fructose. To establish the production AGEs, the fluorescence intensity was measured at *λ*ex = 325 and *λ*em = 440 nm using a Monochromator SpectraMax® M3 plate reader (Molecular Devices, Sunnyvale, CA, USA). To measure the quantities of dityrosine, N′-formyl kynurenine, and kynurenine, fluorescence intensity was measured at *λ*ex = 330, 325, and 365 nm, and *λ*em = 415, 434, and 480 nm [29].

### 2.8. Antioxidant Activity of Q and CH

The compound's antioxidant potential or ability to scavenge reactive oxygen species (ROS) was researched (with modification) as described by [30]. In a 96-well clear plate, Q and CH (10–100 *μ*M) in methanol were added with the blank kept as only methanol. DPPH solution (240 *μ*M) in methanol/tris (1 : 1 v/v), prepared immediately prior to use, was then added to the wells. The same concentrations of Q and CH were added to only methanol/tris (1 : 1 v/v) for control. Using the Monochromator SpectraMax® M3 plate reader (Molecular Devices, Sunnyvale, CA, USA), the absorbance was measured every minute for 10 min at 520 nm.

### 2.9. Direct Relaxation Effect of Q and CH

In accordance with the procedure described earlier, aorta rings were suspended in KHB-filled channels. They were kept at resting tension using the same method previously described, tested for PE contraction and ACh relaxation, and then directly incubated at 37°C for 30 min with the nitric oxide synthase inhibitor, L-NAME (1 mM). A submaximal dose of PE was added to initiate preconstriction; once contraction plateaued, ascending concentrations (10–100 *μ*M) of CH and Q were added. Before the addition of the subsequent concentration, the tissue was given time to reach the relaxation plateau. The vehicle was added to time control channels to eliminate any possible effect of DMSO whose final concentration did not exceed 0.1%. Before termination of the experiment, a single dose of ACh 10^−5^ M was added to all of the channels to achieve the complete aortic relaxation.

### 2.10. Statistical Analysis

One-way analysis of variance (ANOVA) followed by Dunnett's post hoc test was used to analyze the glycation experiment data. Two-way ANOVA followed by Bonferroni post hoc test was used for the vascular reactivity, free radical scavenging activity, and direct relaxation experiments. Unpaired Student's *t*-test was conducted to analyze MetS data. These analyses were carried out using GraphPad Instant software, version 5 (GraphPad Software, Inc., La Jolla, CA, USA). Statistical significance was set at *p* < 0.05. Experimental values were expressed as mean ± SEM (standard error of the mean).

## 3. Results

### 3.1. Effect of High-Fructose High-Salt Diet on MetS Indices

Feeding rats on high-fructose (10% in drinking water) and high-salt diet (3%) for 12 weeks led to development of metabolic syndrome in these animals as indicated by the significant elevations in systolic blood pressure and serum insulin and increase in body weight (Table 1).

### 3.2. Effect of Q and CH on MetS Vascular Dysfunction

**Figure 1:**
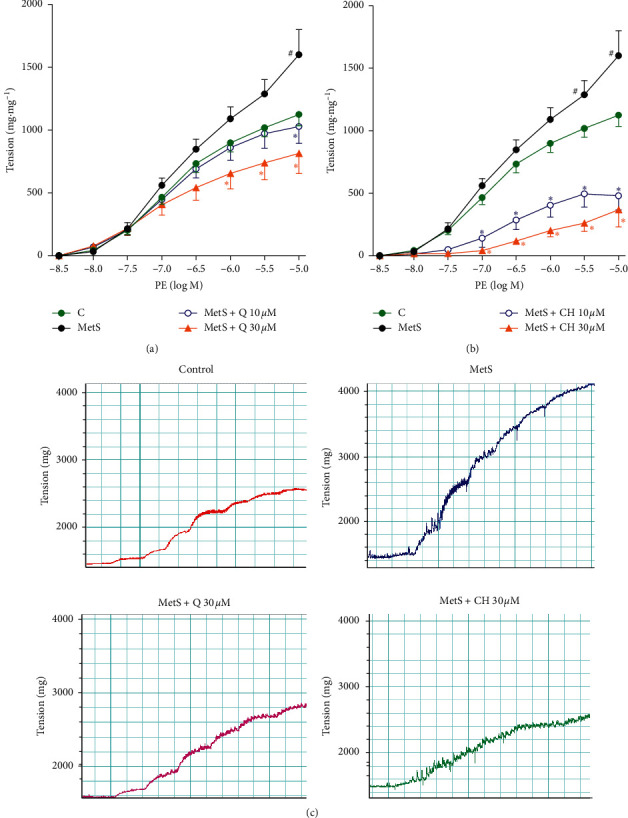
Effect of (a) Q and (b) CH on isolated aorta responsiveness to PE in fructose-induced MetS after incubation with Q or CH at 37°C for 60 minutes. Results are expressed as mean ± SEM (*n* = 6–8). ^#^*p* < 0.05 when compared to control, ^*∗*^*p* < 0.05 when compared to MetS by two-way ANOVA followed by Bonferroni post hoc test. (c) Representative recording charts of control, MetS, MetS + Q 30 *μ*M, and MetS + CH 30 *μ*M experiments.

Compared to control, there was considerably greater vascular contraction in the MetS aorta in response to PE (10^−8^ to 10^−5^ M) (Figure 1). This is indicated by the higher tension in the MetS aorta starting from around 10^−5.5^ M PE concentration and reaching a maximum effect at 142% of the control values. Incubation of the MetS aorta with 10 and 30 *μ*M of Q or CH significantly reduced contraction in a concentration-dependent manner.

**Figure 2:**
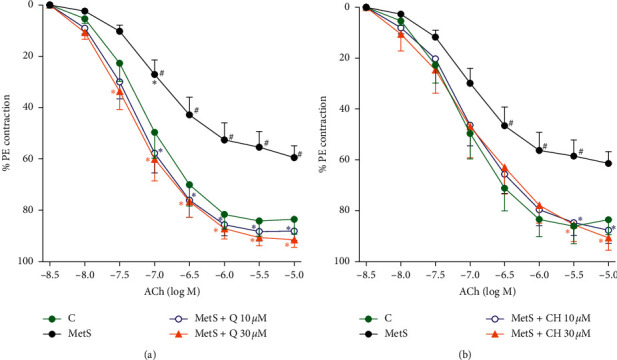
Effect of (a) Q and (b) CH on isolated aorta responsiveness to ACh in fructose-induced MetS after incubation with Q or CH at 37°C for 60 minutes. Results are expressed as mean ± SEM (*n* = 6–8). ^#^*p* < 0.05 when compared to control, ^*∗*^*p* < 0.05 when compared to MetS by two-way ANOVA followed by Bonferroni post hoc test.

As the data in Figures 2(a) and 2(b) demonstrate, vascular relaxation in the MetS aorta was impaired in response to ACh (10^−8^ to 10^−5^ M) compared to the control. There was a significant difference between the MetS aorta and the control, with the effect appearing at approximately 10^−6.5^ M ACh and reaching a maximum relaxation at 72% of the control values. Incubating the MetS aorta with 10 and 30 *μ*M of Q or CH corrected the attenuated vasorelaxation to control levels. The graphs show that these concentrations of the compounds were similarly effective in lowering the aorta tension during relaxation.

### 3.3. Effect of Q and CH on MG-Induced Vascular Dysfunction

**Figure 3:**
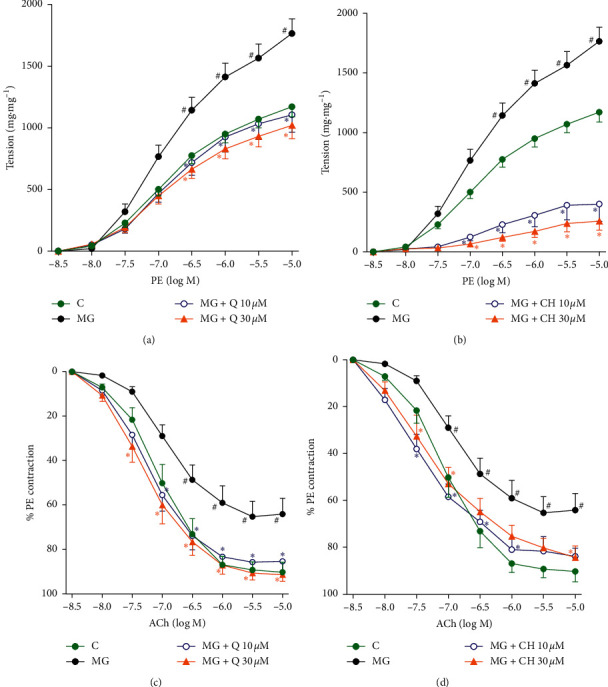
Effect of (a) Q and (b) CH on isolated normal aorta responsiveness to PE after incubation with MG (100 *μ*M) in the presence and absence of Q or CH at 37°C for 60 minutes. Effect of (c) Q and (d) CH on isolated normal aorta responsiveness to ACh after incubation with MG (100 *μ*M) in the presence and absence of Q or CH at 37°C for 60 minutes. Results are expressed as mean ± SEM (*n* = 6–8). ^#^*p* < 0.05 when compared to control, ^*∗*^*p* < 0.05 when compared to MG by two-way ANOVA followed by Bonferroni post hoc test.

As Figures 3(a) and 3(b) show, the addition of MG to the aorta caused exaggerated vascular contraction in response to PE (10^−8^ to 10^−5^ M) when compared to control. This is demonstrated by the tension in MG-treated aorta being significantly higher, starting from 10^−6.5^ M PE concentration. Both concentrations of Q and CH (10 and 30 *μ*M) significantly reduced the extent of contraction, returning it back to normal and, in the case of CH, even below that of the control.

Figures 3(c) and 3(d) show that, following incubation with MG, smooth muscle dilatation of the aorta in response to ACh (10^−8^ to 10^−5^ M) was impaired compared to control. As portrayed in Figures 3(c) and 3(d), there was a significant difference between the MG-treated aorta and the control during vascular relaxation, with the effect appearing at approximately 10^−7^ M ACh. Both concentrations of Q and CH (10 and 30 *μ*M) were able to ameliorate this impaired vasorelaxation, restoring it to values that were comparable to those of control.

### 3.4. Effect of Q and CH on AGE Production

**Figure 4:**
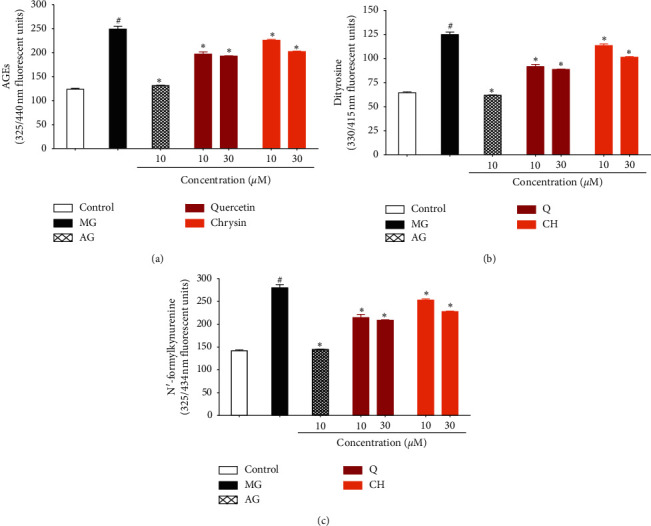
Effect of Q and CH on (a) AGE, (b) dityrosine, and (c) N-formyl kynurenine production upon incubation of BSA (10 mg/ml) with MG (50 mM) at 37°C for one hour. Control is a reaction mixture including only BSA while the MG reaction mixture consists of BSA with MG. AG was used as positive control. Results are expressed as mean ± SEM (*n* = 3). ^#^*p* < 0.05 when compared to control, ^#^*p* < 0.05 when compared to MG by one-way ANOVA followed by Dunnett's post hoc test.

Figures 4(a)–4(c) show that, compared with the control, incubation of BSA (10 mg/ml) with MG (50 mM) resulted in a significant increase in AGE production, as well as the protein oxidation products, dityrosine, kynurenine, and N-formyl kynurenine. Addition of AG (1 mM) in the reaction mixture significantly suppressed the levels of AGEs and the protein oxidation products. Both concentrations of Q and CH were significantly able to inhibit the formation of MG-mediated AGEs and resultant protein oxidation in a concentration-dependent manner.

**Figure 5:**
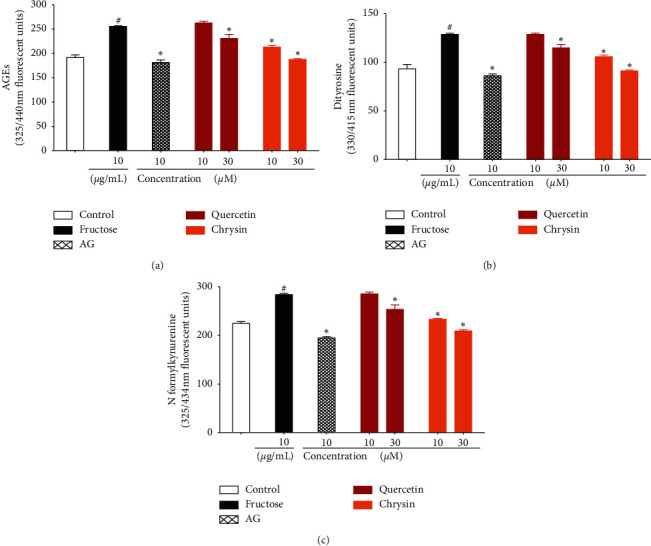
Effect of Q and CH on (a) AGE, (b) dityrosine, and (c) N-formyl kynurenine production upon incubation of BSA (10 mg/ml) with F (50 mM) at 37°C for two weeks. Control is a reaction mixture including only BSA while the F reaction mixture consists of BSA with F. AG was used as positive control. Results are expressed as mean ± SEM (*n* = 3). ^#^*p* < 0.05 when compared to control, ^*∗*^*p* < 0.05 when compared to F by one-way ANOVA followed by Dunnett's post hoc test.

A similar pattern of results is seen for the fructose-mediated glycation reaction (Figures 5(a)–5(c)). Incubating BSA with F (50 mM) resulted in a significant increase in AGE and protein oxidation products compared with the control. This was significantly inhibited by AG (1 mM). With the exception of 10 *μ*M of Q, levels of AGE and protein oxidation were significantly reduced by all concentrations of CH and Q.

### 3.5. Free-Radical Scavenging Activity of Q and CH

**Figure 6:**
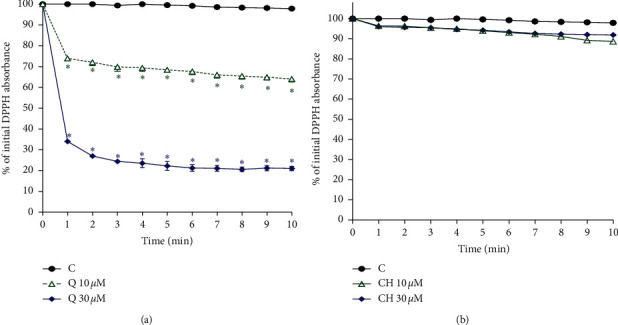
Effect of (a) Q and (b) CH on ROS production as initiated by DPPH (240 *μ*M). Control is a reaction mixture including only DPPH. Results are expressed as mean ± SEM (*n* = 3). ^*∗*^*p* < 0.05 when compared to each corresponding control by two-way ANOVA followed by Bonferroni post hoc test.

Figures 6(a) and 6(b) display the results of the 10 min reaction between DPPH and the natural compounds. The results indicate that only Q possessed DPPH free radical scavenging activity, which is translated into an antioxidant effect.  Figure 6(a) shows that both concentrations of Q operate in a concentration-dependent manner significantly influencing antioxidant activity. Furthermore, the graph reveals that that the reaction took place within the first few minutes and then reached a plateau. The time taken to reach that plateau varied according to the concentration used. As expected, a faster reaction was seen with the 30 *μ*M Q, lasting for 6 minutes, compared to a 10 minute reaction time when Q 10 *μ*M was added.

### 3.6. Direct Relaxation Effect of Q and CH

**Figure 7:**
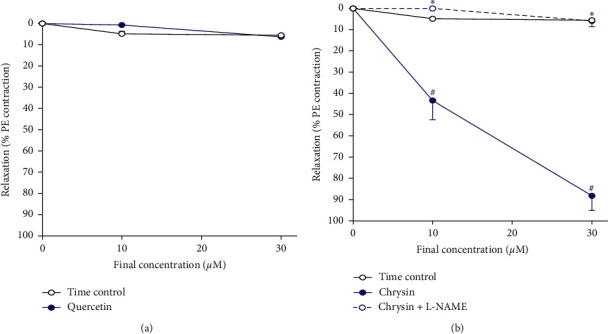
Direct vasorelaxation effect of the compounds (a) Q and (b) CH on PE-precontracted isolated aorta with and without incubation with L- NAME (1 mM) at 37°C for 30 minutes. Results are expressed as mean ± SEM (*n* = 6–8). ^*∗*^*p* < 0.05 when compared to control by two-way ANOVA followed by Bonferroni post hoc test.

Following a single dose of PE (10^−5^ M) to induce contraction, the addition of cumulative concentrations of Q (at 10 and 30 *μ*M) to the aorta did not have any effect (Figure 7(a)). However, addition of cumulative concentrations of CH (at 10 and 30 *μ*M) to the aorta brought about a decrease in tension and hence concentration-dependent vasodilation (*p* < 0.05, Figure 7(b)). CH produced potent vasodilation that reached 90% relaxation at concentration of 30 *μ*M of CH. In addition, the aorta incubated with L-NAME (1 mM) completely blocked the mentioned CH potent vasodilation (*p* < 0.05, Figure 7(b)).

## 4. Discussion

The purpose of this study was to investigate whether Q and CH possess vasculoprotective effects to ameliorate vascular damage commonly seen in MetS and to determine possible mechanisms of action. To our knowledge, this is the first study to investigate the direct vasculoprotective effects of the natural compounds Q and CH on MetS aorta and the role of AGE inhibition in their effects. The results showed that Q and CH protect against MetS associated vascular dysfunction. The common effect of Q and CH compounds was to (1) interfere with AGEs-induced exaggerated vasoconstriction and impaired vasodilation and (2) significantly inhibit AGEs forming in a dose-dependent manner. In addition, Q had extra free radical scavenging activity, while CH showed nitric oxide-dependent vasodilation.

Vascular dysfunction is associated with several MetS-related factors, the most important of which are the advanced glycation end products (AGEs) [31]. Such vascular dysfunction is characterized by increased vasoconstriction and attenuated dilatation [15]. Other than MetS, vascular dysfunction can be directly induced *in vitro* with the use of the glycation intermediate methylglyoxal (MG). Being a highly reactive sugar derivative, MG can result in acute production of AGEs, which is largely responsible for vascular damage. This finding is in agreement with that of Dhar et al. [32] who proved that MG results in vascular dysfunction characterized by attenuated ACh-induced aortic relaxation.

The results show that Q and CH significantly alleviated the exaggerated vasoconstriction of the aortic rings and attenuated ACh-induced vasodilation found in MetS aortae. This finding is consistent with that of Sánchez et al. [33] who demonstrated that *in vivo* treatment with Q in hypertensive rats enhanced aortic vasodilation. In addition, El‐Bassossy et al. [34] also proved the ability of CH treatment to improve the exaggerated vasoconstriction in insulin-resistant rats.

The effect on AGEs-induced vascular damage was investigated as a possible mechanism of action of Q and CH. In this regard, both Q and CH inhibited MG-induced exaggerated vasoconstriction and impaired vasodilation in a very similar way to that observed in MetS aortae. This suggests that AGEs can be a common important pathway for Q and CH vascular protection effect.

The effect on AGEs formation was studied in order to further investigate the effect of Q and CH on different sides of the AGEs pathways. The current study showed that both Q and CH significantly inhibit both fructose- and MG-produced AGEs in a concentration-dependent manner. One hypothesized mechanism for Q's antiglycation action is that the compound has a polyphenolic structure, enabling it to scavenge free radicals. The structure, which is abundant with hydroxyl groups, is essential to the antioxidant effect shown by the compounds [35]. Such compounds donate a hydrogen atom to reduce free radicals, hence preventing protein oxidation, a main step in AGE formation. This is further proven by the strong antioxidant activity of Q in its reaction with the DPPH free radical. This is consistent with several previously conducted studies, which have researched the antiglycation activity of Q [36–38].

Regarding the flavonoid CH, its inability to scavenge DPPH free radicals has been investigated by Kang et al. [39], who reported that CH is missing two hydroxyl groups, which are essential for the compound (flavone) to exhibit antioxidant activity. This is further proven by Naso et al. [40], who stated that CH was unable to diminish the levels of DPPH radicals. However, despite CH possessing lower antioxidant potential than flavonoids such as Q, it is still able to prevent protein glycation significantly. This finding coincides with that of Matsuda et al. [41] who stated that various flavonoids with strong AGE formation inhibition may be poor scavengers of DPPH radicals. A suggested mechanism of action of CH in inhibiting the formation of AGEs could be the strong affinity of the flavone to BSA, which prevents the protein's glycation [42]. The ability of CH to decrease AGE levels has also been demonstrated *in vivo* where the flavonoid significantly inhibited the increase in serum AGEs in diabetic animals [34].

A mechanism of action for such an effect might be that, in response to CH and Q, NO is released from endothelial tissue, directly causing PE-precontracted aorta to relax significantly. Evidence in support of this NO-dependent mechanism comes from the compounds' direct relaxation effect on precontracted aorta, which was significantly reduced in the presence of the eNOS inhibitor L-NAME. A closer observation of the results shows that both the MG-treated aorta and the CH-treated MetS aorta display vasoconstriction much lower than that seen in control animals. This effect was unique for CH rather than Q. Furthermore, L-NAME inhibited a direct relaxation effect of CH more than that of Q. Both these findings may suggest that CH in particular causes potent eNOS stimulation producing greater levels of NO when compared to Q. This is in accordance with the findings of Villar et al. [43] who concluded that CH, unlike several other flavonoids, brought about aortic relaxation *in vitro* mainly through endothelium-dependent methods involving increased endothelial NO production. Duarte et al. [44] further argued that CH may in fact induce vascular relaxation in isolated aorta through additional NO-related mechanisms other than eNOS stimulation. The study proved that CH was able to improve the response of the aorta to authentic NO through scavenging superoxide anions (O2•-) which broke down NO. In addition, CH also potentiated the cGMP pathway, signaling cascade involved in NO-induced vasodilation. This was proven by previous studies which reported an increase in cGMP accumulation in isolated rat aorta by CH (Villar et al. 2005).

The results of this study prove that, apart from the NO-releasing mechanism, both Q and CH protect aorta sections from vascular damage by preventing AGEs from forming. The AGEs alter the structure of proteins and their functions. In the case of blood vessels, glycated collagen results in reduced vessel elasticity [45]. In addition, elevated levels of AGE increase oxidative stress, activate inflammatory mediators, alter the lipid profile, and quench NO, all of which contribute to endothelial damage [46].

The obvious effect of these compounds on ameliorating vascular dysfunction, as proven by this study, can be used as a basis for future research. In addition, the possible effects that the compounds may have in vivo, when added to the diet, and the possible improvement in the same parameters measured can be further investigated.

## 5. Conclusions

The current study shows that the natural flavonoid compounds Q and CH have direct vasculoprotective effects on isolated thoracic aorta from MetS rats. This is demonstrated by the compounds' ability to ameliorate the exaggerated vasoconstriction and attenuated vasodilation typical of MetS vascular dysfunction. This effect can be attributed to the compounds' ability to reduce AGE production as well as the concomitant protein oxidation products. In addition, there is significant dependence on NO-mediated mechanisms and, in the case of Q, antioxidant activity, which contribute to the vascular protection.

## Figures and Tables

**Table 1:** Effect of high-fructose (10%) high-salt (3%) feeding for 12 weeks on the increase in systolic blood pressure (SBP), body weight, and serum insulin in rats.

Treatment	SBP (mmHg)	Body weight (g)	Serum insulin (ng/dl)
Control	115.2 ± 3.9	285.8 ± 10.4	2.32 ± 0.33
MetS	134.5^*∗*^ ± 6.7	356.2^*∗*^ ± 16.8	6.93^*∗*^ ± 0.97

Values are expressed as mean ± SEM; ^*∗*^*p* < 0.05, compared with the corresponding control group values using unpaired *t*-test. *N* = 6 rats.

## Data Availability

The data used to support the findings of this study are available from the corresponding author upon request.

## Abbreviations

ACh: Acetylcholine
AGEs: Advanced glycation end products
AG: Aminoguanidine
ANOVA: Analysis of variance
BSA: Bovine serum albumin
Q: Quercetin
CH: Chrysin
CVD: Cardiovascular diseases
DPPH: Diphenyl-2-picrylhydrazyl
L-NAME: Nω-Nitro-L-arginine methyl ester hydrochloride
MetS: Metabolic syndrome
PE: Phenylephrine
MG: Methylglyoxal
NO: Nitric oxide
PBS: Phosphate buffer saline
T2DM: Type 2 diabetes mellitus
SBP: Systolic blood pressure.
